# A rarely described Kimura’s disease of the breast

**DOI:** 10.1259/bjrcr.20220160

**Published:** 2023-09-12

**Authors:** Eman A. Almohawes, Yasser Zakareia, Zainab A. Dumiati, Wael A. Zaki, Khalid M. Alfudhili

**Affiliations:** 1 Medical Imaging Department, Security Forces Hospital-Dammam, Dammam, Saudi Arabia; 2 Medical Imaging Department, Radiology Registrar, Prince Sultan Armed Forces Hospital, Al Madinah, Saudi Arabia; 3 General Surgery Department, Consultant Surgeon, Security Forces Hospital-Dammam, Dammam, Saudi Arabia

## Abstract

Kimura’s disease is a rare chronic inflammatory disease of unknown aetiology. The majority of cases are reported in middle-aged Asian males and rarely seen in other ethnicities. Patients classically present with chronic single or multiple painless subcutaneous masses, lymphadenopathies, hypereosinophilia and elevated serum immunoglobulin E. The disease usually affects the head and neck area, however, rare involvement of other locations such as axilla, upper limbs, popliteal region and abdomen has been reported. Here, we report a rarely encountered Kimura’s disease of the breast and axillary lymph nodes in a middle-aged female. In this report, we will describe the main clinical, radiological and pathological features to raise the awareness about this indolent disease.

## Clinical presentation

A 58-year-old post-menopausal middle-eastern lady was referred to the General Surgery Outpatients Clinic from the Haemodialysis Unit in our hospital after noticing right breast painless enlargement over 10 months. Patient is known to have diabetes mellitus, hypertension, and end-stage renal disease on haemodialysis through left internal jugular vein central dialysis catheter. She had no history of fever, night sweats or weight loss. There was no history of previous breast pathology or family history of breast or ovarian cancers. She had undergone three caesarean sections and had used oral contraceptives for 1 year. Clinical examination showed diffuse non-tender swelling of the right breast and enlarged right axillary palpable, firm, non-fixed and non-tender lymph nodes. There was no skin redness or discolouration and no palpable breast mass. Routine blood tests revealed peripheral eosinophilia of 14% (normal, 1–3%). Additional immunologic blood test was done to investigate peripheral eosinophilia and showed a high serum immunoglobulin E (IgE) (2153 UI/mL; normal, 80–120 UI/mL). Based on clinical presentation, the initial differential diagnosis included primary breast carcinoma/sarcoma or metastasis, breast lymphoma (primary or secondary), inflammatory disorders, superior vena cava obstruction by thrombosis or mediastinal mass or non-puerperal mastitis.

## Imaging findings

**Figure 1.**
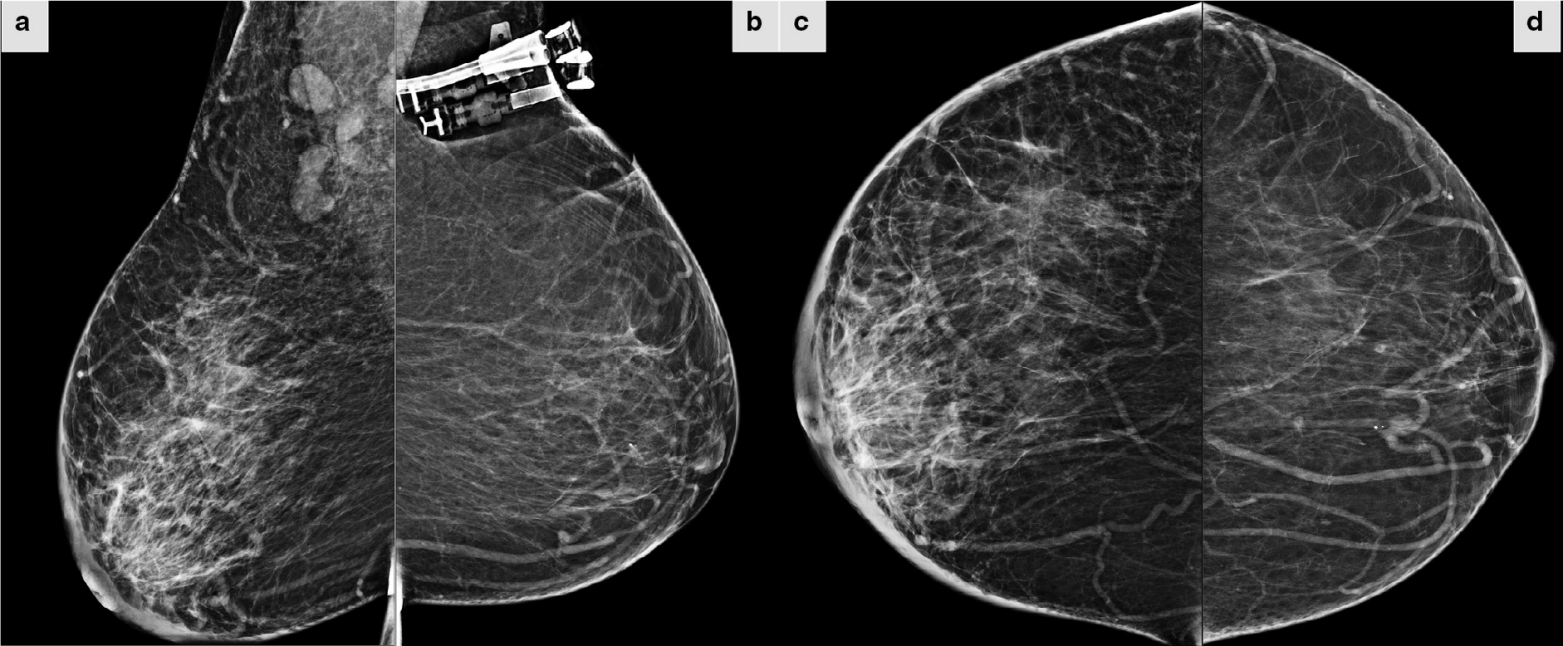
Digital mammography of the right breast, (**a**) CC and (**c**) MLO views, shows diffuse subcutaneous fat infiltration associated with increased trabeculation and dermal thickening are consistent with oedema, however, no definite mass, parenchymal distortion or suspicious microcalcifcation. Right axilla shows multiple abnormally enlarged lymph nodes with thickened cortex. Left breast (**b, d**) mammography CC and MLO views demonstrate left haemodialysis catheter and predominantly fatty breast parenchyma with scattered remaining few fibroglandular densities without mass, architectural distortion or suspicious group of microcalcification. CC, craniocaudal; MLO, mediolateral oblique.

**Figure 2.**
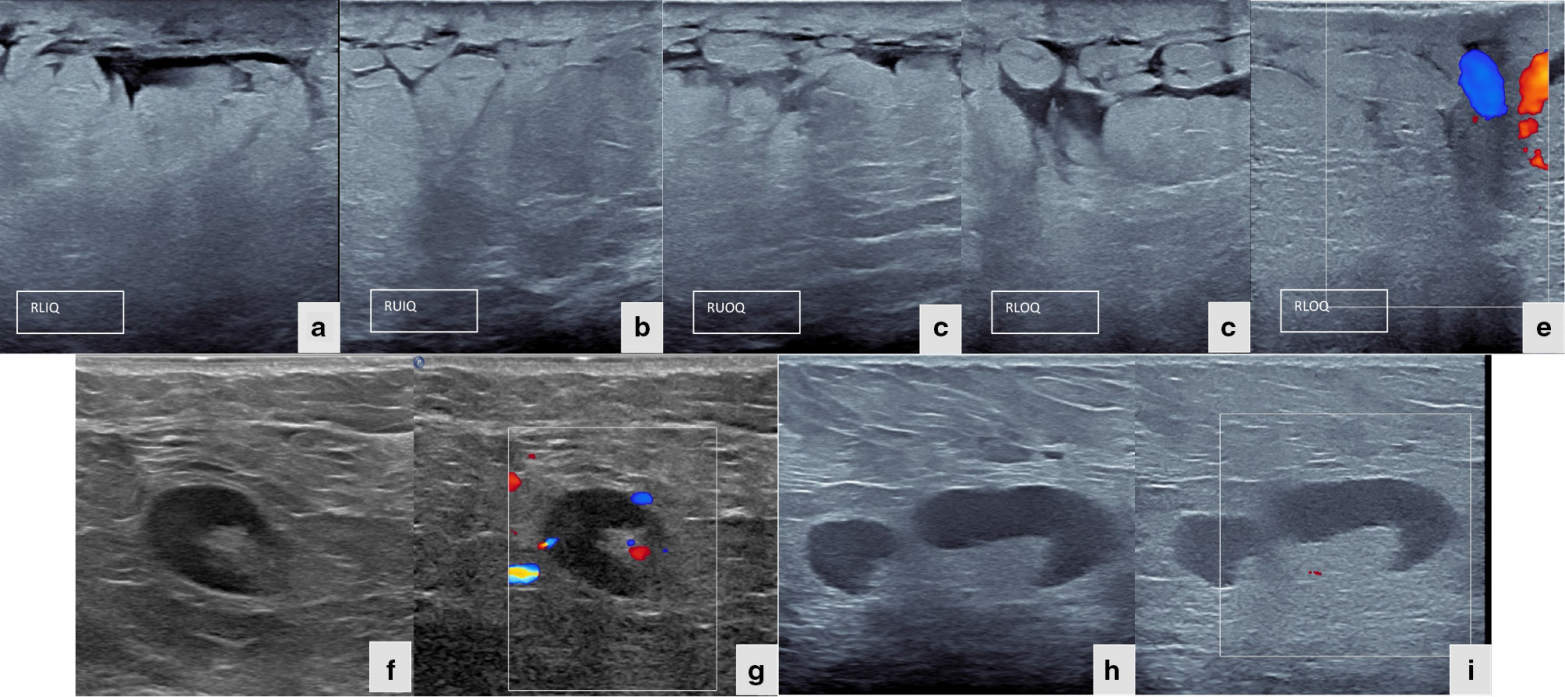
(a–e) Ultrasound and colour doppler of the four quadrants of the right breast shows skin thickening with oedematous changes involving the fat lobules of the breast and mildly increase blood flow. No evidence of ductal dilatation or mass seen. (f–i) Ultrasound and doppler ultrasound of the right axilla shows multiple enlarged lymph nodes with diffuse thicken cortex, however, fatty hilum is preserved with mild increased of blood flow.

**Figure 3.**
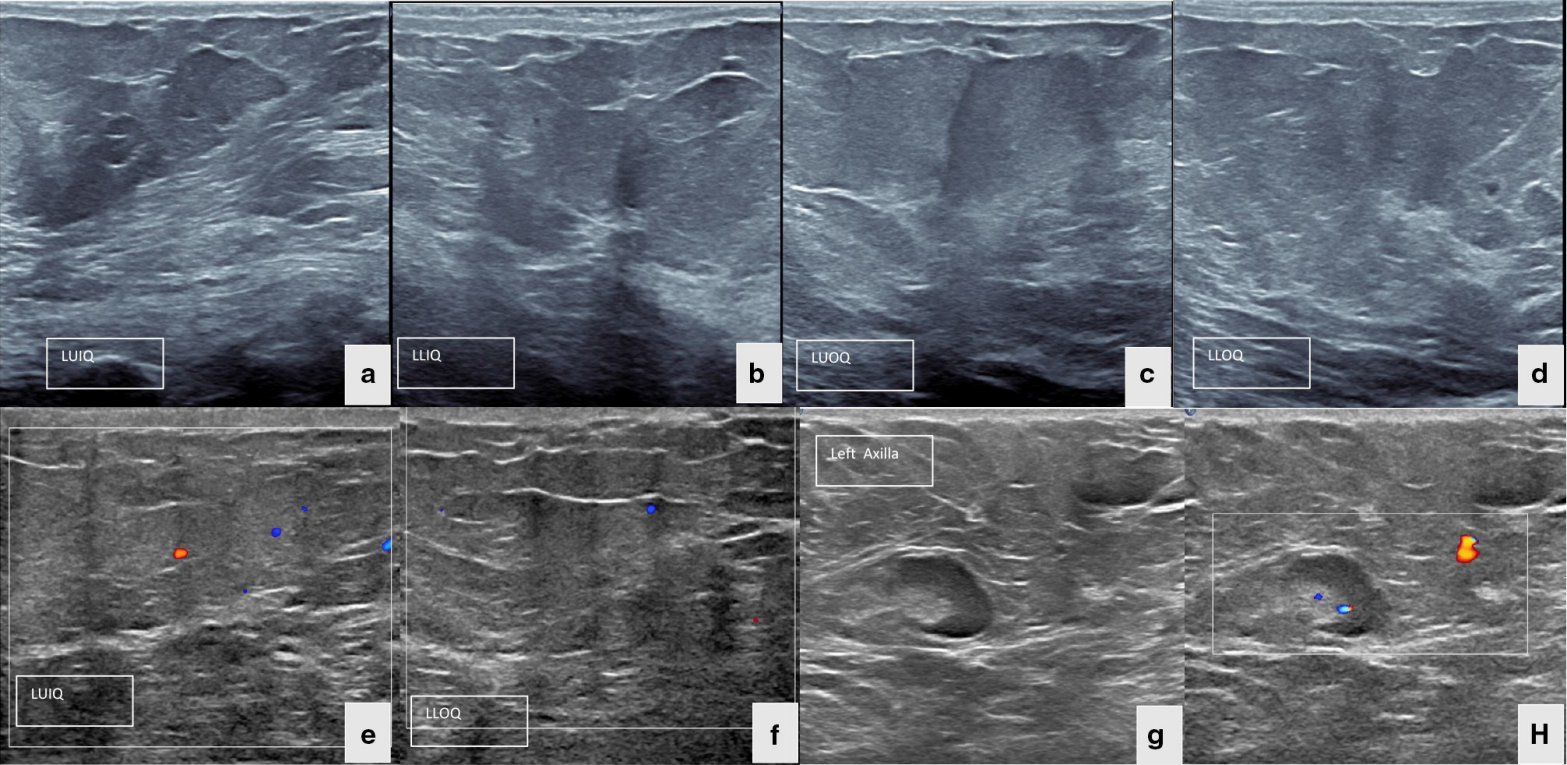
(a–h) Ultrasound and colour doppler of the four quadrants of the left breast demonstrate normal parenchyma and blood flow.

**Figure 4.**
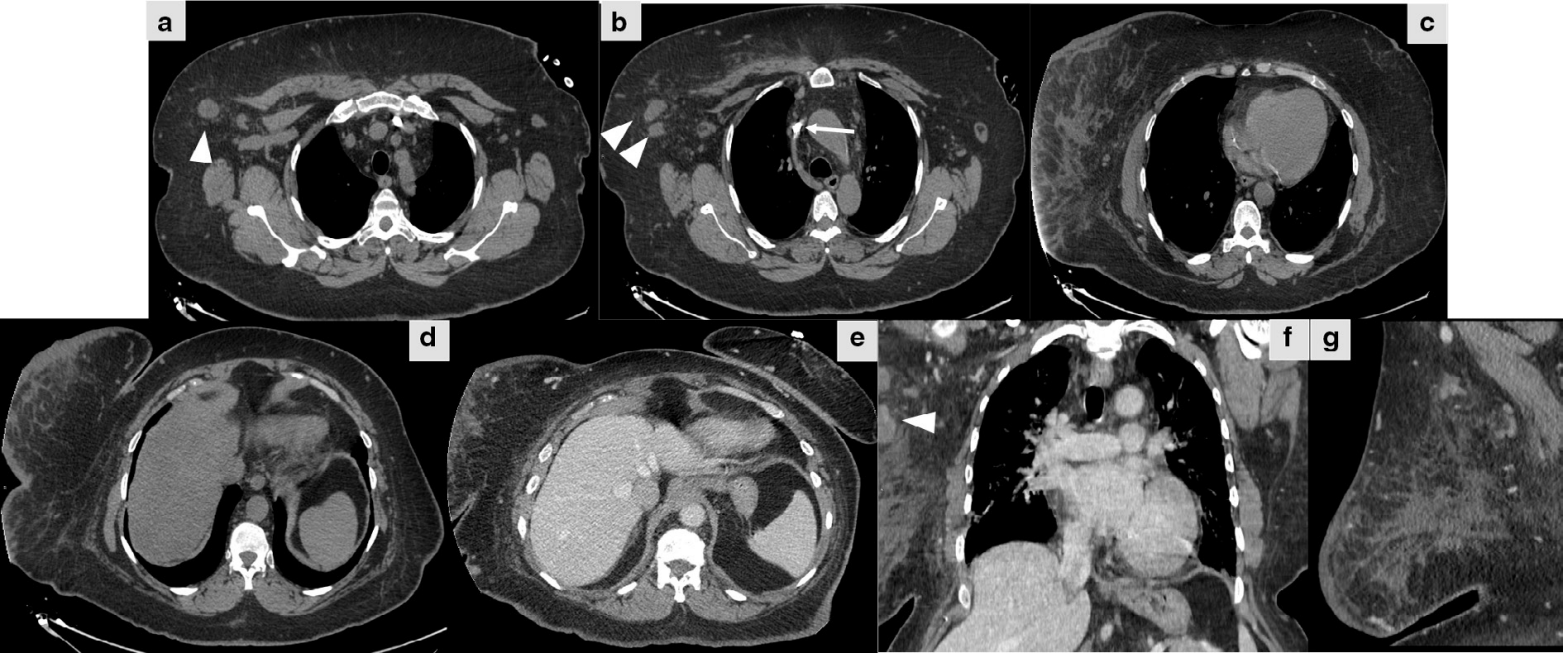
Non-enhanced CT scan of the chest, axial (a–e), coronal (**f**) and sagittal (**g**) images reveals a partially seen diffuse enlargement of the right breast with skin thickening and diffuse fat stranding and right multiple axillary lymphadenopathy (arrowheads). (e–g) Post-contrast CT coronal image shows no enhancement of the breast tissue. Partially seen Permacath inserted into left internal jugular vein (arrow) for heamodialysis.

Patient was referred to Breast Imaging Unit in Radiology Department for mammography and breast ultrasound. Mammography demonstrated right breast enlargement with dense parenchyma, dermal thickening, and right axillary lymphadenopathy without suspicious mass lesion or microcalcifications (Figure 1). On the other hand, right breast ultrasound revealed thickening of the skin and multiple parenchymal hypoechoic areas consistent with fat oedematous changes without suspicious mass. Multiple enlarged ipsilateral axillary lymph nodes showing thickened cortex and eccentric fatty hilae are also noted. Colour doppler ultrasound showed mild increased vascularity in the affected breast and axillary lymph nodes (Figure 2). Left breast was normal (Figure 3). Then, contrast- enhanced chest CT scan was performed to rule out mediastinal pathology and evaluate lung parenchyma. The exam revealed diffuse enlargement of the right breast, that was partially included in the field of view of the scan due to big size, with extensive parenchymal fat stranding and dermal thickening accompanied by multiple right axillary lymphadenopathies. No suspicious breast mass lesion, calcification or abnormal enhancement in the scanned part of the breast. No pulmonary mass or nodule, nor mediastinal mass or lymphadenopathy (Figure 4). Breast MRI with gadolinium i.v. contrast was recommended for evaluation of the right breast abnormality. However, patient refused to proceed with doing MRI because of claustrophobia and anxiety about the possible nephrogenic systemic fibrosis complication of gadolinium contrast medium to patients with chronic kidney disease on haemodialysis.

**Figure 5.**
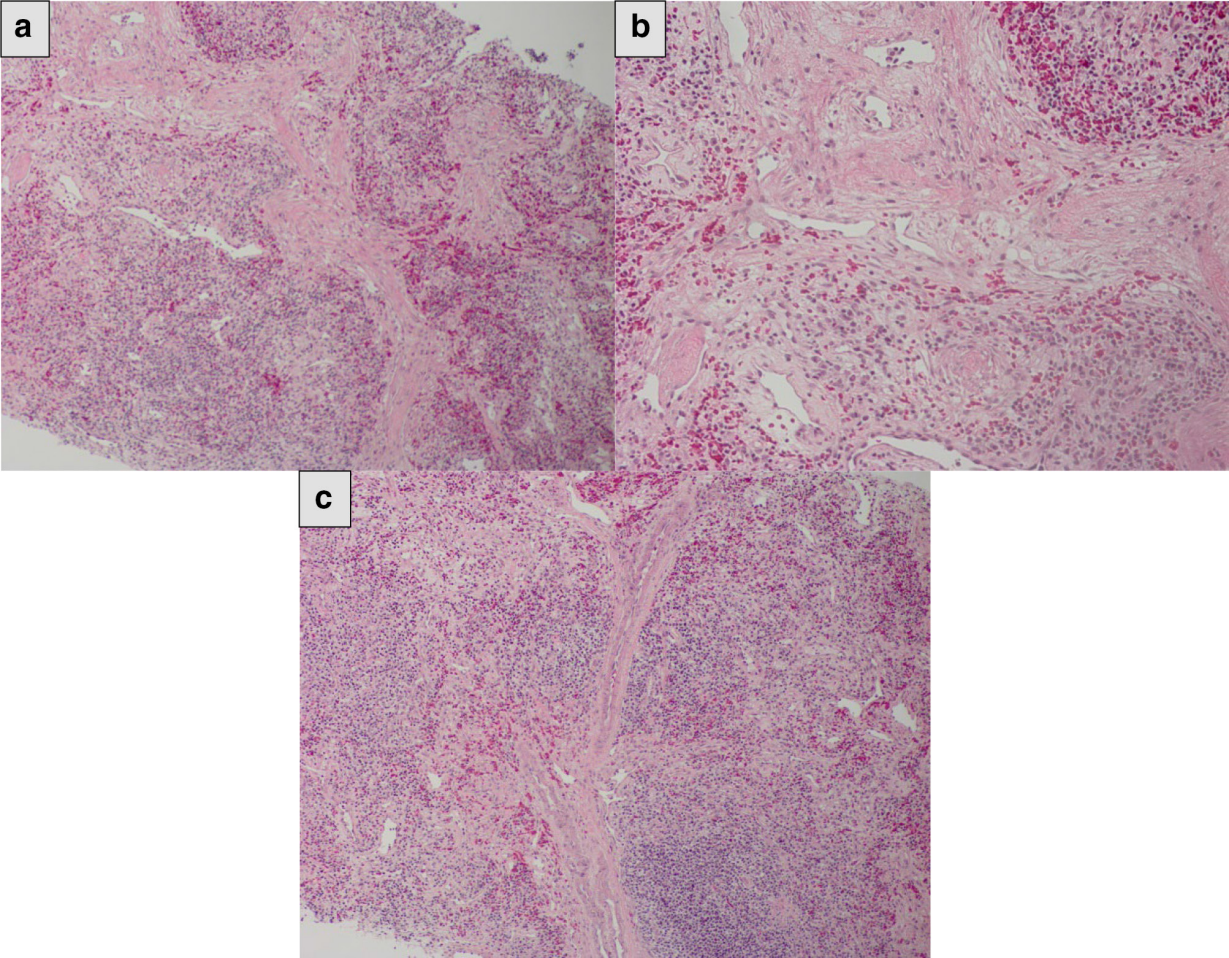
Image (**a, b**) (HE x 40): Proliferation of vascular structures and hyperplastic follicles with accompanying mixed inflammatory infiltrate. Image c (HE x100): Mixed inflammatory infiltrate consisting of eosinophils plasma cells and lymphocytes.

Depending on clinical and radiological findings, right breast malignant pathology was suspected. Utrasound-guided trucut needle biopsy of the affected axillary lymph nodes was performed and the tissue samples was insufficient for a final diagnosis. 3 weeks later, in a different appointment, the biopsy was repeated in a different affected lymph node and again it was inconclusive. Subsequently, surgical excision of the affected axillary lymph nodes was done and microscopic examination showed multiple lymphoid follicles associated with prominent germinal centres, fibrosis, and inflammatory infiltrates of eosinophils, lymphocytes, and histiocytes, with a predominance of eosinophils. Eosinophilic abscesses and a few irregularly shaped enlarged blood vessels were also observed (Figure 5).

## Management and follow-up

**Figure 6.**
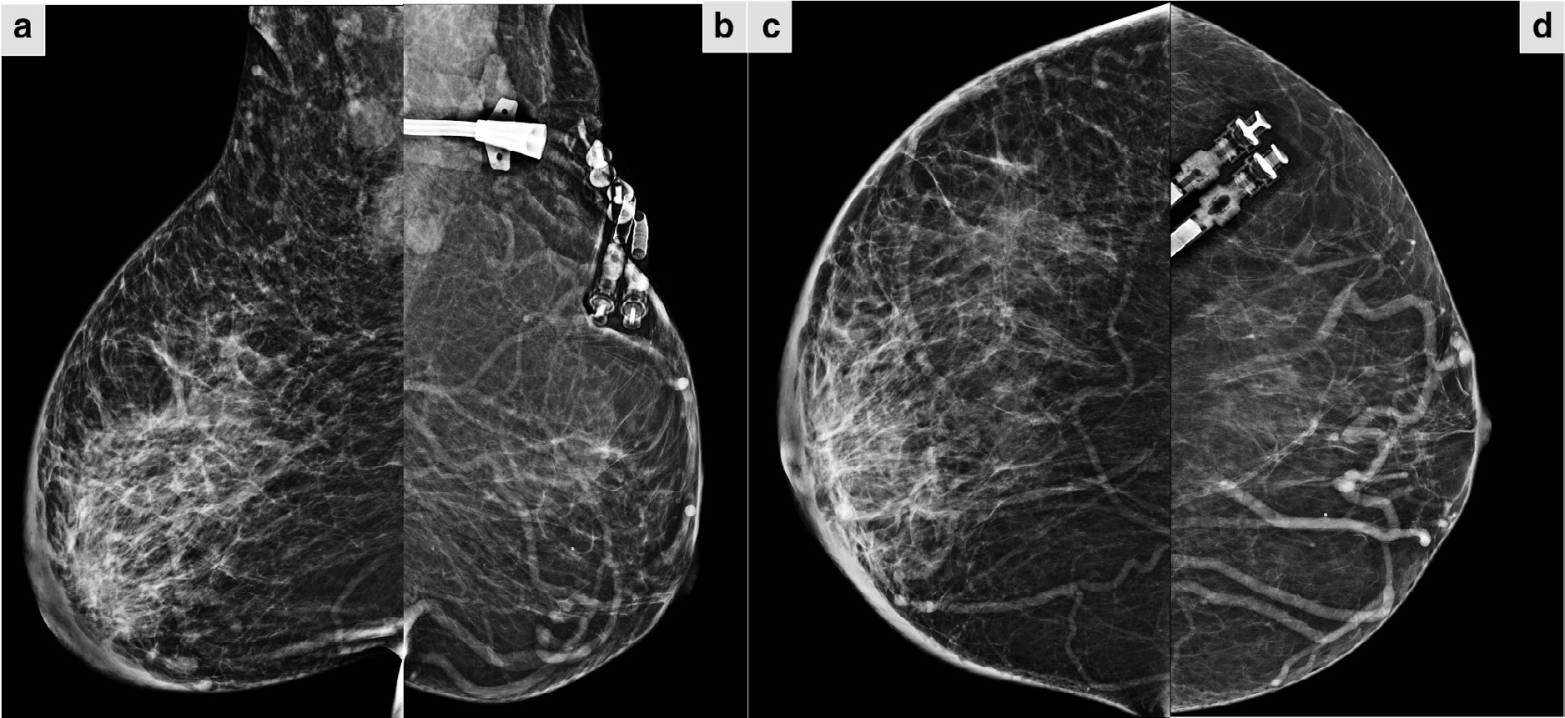
12-month follow-up mammography of both breasts, MLO and CC views, showing unchanged enlarged oedematous right breast and right axillary adenopathy (**a, c**). CC, craniocaudal; MLO, mediolateral oblique.

A final diagnosis of Kimura’s disease (KD) of the breast was made based on the pathological features and presence of peripheral hypereosinophilia and high IgE. Treatment options including close observation, oral steroid or referring to a tertiary hospital for immunosuppressive therapy, radiotherapy and surgical excision were discussed with the patient. Because she was asymptomatic, the treating team decided in accordance with patient’s wish to observe her closely. During follow-up period, count of eosinophil and serum IgE remained high, ranging around 11–14% and 2016–2125 UI/mL respectively. 12 months after initial diagnosis, follow-up mammography showed almost unchanged right breast abnormality with right axillary lymphadenopathy (Figure 6). Consequently, she was given a high dose of oral prednisolone 60 mg daily for 3 months with gradual tapering. Following steroid course, right breast enlargement reduced in size and count of eosinophil and IgE level showed significant improvement at (6%) and (487UI/mL) respectively. Follow-up breast ultrasound demonstrated improvement of the right breast oedema. As of this writing, patient is on long-term follow-up with uneventful recovery and she has three routine haemodialysis sessions per week in our hospital, annual follow-up mammography and laboratory tests quarterly for 1 year.

## Discussion

KD is a rare chronic inflammatory disorder initially described by Kimm and Szeto in 1937 under the name (eosinophilic hyperplastic lymphogranuloma).^1^ Later in 1948, Kimura et al characterised and classified the pathological features of this disease.^2^ The disease is more prevalent in Asian (Chinese, Japanese, and Southeast Asian) males aged 10–40 years. A few sporadic cases, however, have been documented in Western populations.^1^ The vast majority of the affected regions are in the head and neck, but, involvement of unusual sites such as axilla, groin, limbs and abdomen has also been reported.^1^ Breast, as observed in our patient, is an unusual location for disease involvement and, to the best of our knowledge, the first case of KD in the breast was published in the medical literature in 2016 by Kakkar et al who reported a 40-year-old female with breast KD presented with painful breast lump and axillary lymphadenopathy.^3^ Later in 2020, another case report described a case of KD of the breast in a 50-year-old Asian female, which had bilateral multifocal breasts masses and bilateral axillary lymphadenopathy.^4^ In comparison to the previously reported cases, the distinct presentation of our patient was painless diffuse breast enlargement and axillary lymphadenopathy without palpable breast mass or focal lesion.

Clinically, most patients with KD present with chronic painless, firm, single or multiple subcutaneous masses in the head and neck associated with regional lymphadenopathy and parotid and submandibular swelling.^5^ Long-standing isolated lymphadenopathy may also be the only initial clinical presentation.^5^ These manifestations are almost always accompanied by distinctive laboratory findings, including peripheral eosinophilia and elevated serum IgE, which are crucial to exclude other diagnoses.^1,5^ Although the process of KD development is still not clear, peripheral eosinophilia and high serum IgE imply that the disease could be related to an aberrant hypersensitivity reaction initiated by unknown antigenic stimulus which may include a viral infection, arthropod infestation or neoplasm.^3^ Moreover, immune-complex-mediated renal impairment, which may progress to nephrotic syndrome, has been reported in approximately 12% of patients.^1,6^ Widespread vascular thrombosis with KD was also described and attributed to hypercoagulable state promoted by either nephrotic syndrome or hypereosinophilia.^7,8^ Nevertheless, Lee et al reported a case of KD in a Korean male with normal renal function test who presented with massive thrombosis resulting in infarction of the small bowel and, surprisingly, his serum eosinophil count was not elevated when he developed thrombotic infarction.^8^ Accordingly, the underlying mechanism of the hypercoagulability accompanying KD thus far is debatable and requires more investigation.

Radiological findings of KD are non-specific and can be similar to those of other benign or malignant conditions; however, medical imaging is useful in assessing disease extent. MRI is the modality of choice for assessing lesion characteristics because of its higher soft tissue resolution.^1^ Due to variable degree of fibrosis and vascularity, soft tissue lesions may appear well-demarcated or infiltrative, iso- or hyperintense to muscles on *T*_1_W images, hyperintense on *T*_2_W and short-tau inversion recovery images, and enhanced homogeneously or heterogeneously on post-contrast images.^1^ In contrast, the affected lymph nodes usually show homogeneous enhancement without necrosis. Flow voids in lesions, indicating vascular proliferation, have also been observed.^1,9^ Notwithstanding the diffusivity of lesions on diffusion images has not been thoroughly studied; yet Wang et al reported that apparent diffusion coefficient (ADC) values were higher in the affected parotid gland than in the normal contralateral gland.^10^ This feature can help in distinguishing KD lesions from malignant parotid lesions, which typically show low ADC values. Furthermore, ADC values in lymphadenopathies were lower than those in unaffected lymph nodes.^10^ On CT scans, lesions may appear hypodense on pre-contrast CT images and homogeneously or heterogeneously enhanced on post-contrast images.^1,9^ Ultrasound examination usually demonstrates swollen salivary glands with heterogeneous echotexture with a rich blood flow on colour doppler.^1^ Hypoechoic subcutaneous vascularised masses with ill-defined borders and irregular shapes may also be observed. The affected lymph nodes appear enlarged with fatty hypervascular hilae (Table 1).^1,4,11^

**Table 1.** Imaging characteristics of Kimura’s disease

Modality	Imaging characteristics
MRI	Well-demarcated or infiltrative lesions. Isointense or hyperintense to muscles on *T*_1_W images. Hyperintense on *T*_2_W and STIR images. Homogeneous or heterogeneous enhancement on post-contrast images. Affected lymph nodes usually show homogeneous enhancement without necrosis. Flow voids in lesions, indicating vascular proliferation. High ADC values in the lesions. Low ADC values in affected lymph nodes.
CT scan	Lesions appear hypodense on pre-contrast CT images. Homogeneous or heterogeneous enhancement on post-contrast images.
Ultrasound and colour doppler	Affected area shows heterogeneous echotexture with rich blood flow on colour doppler. Hypoechoic subcutaneous vascularised masses with ill-defined borders and irregular shapes. The affected lymph nodes appear enlarged with fatty hypervascular hilae.

ADC, apparent diffusion coefficient; STIR, short-tau inversion recovery.

Pathological assessment of the surgical specimen biopsy is the gold-standard for the definitive diagnosis of KD.^1^ Most lesions microscopically demonstrate reactive follicular hyperplasia, many prominent lymphoid follicles, and enlarged germinal centres with massive eosinophilic infiltration, a variable degree of fibrosis, and high vascularity. Eosinophilic micro-abscesses are observed in a few cases.^1,2^

The diagnosis of KD is challenging, and misdiagnosis as malignancy is common due to the lack of pathognomonic features. Therefore, clinical presentation, laboratory findings, radiological characteristics and pathological diagnosis are necessary for accurate diagnosis. Although KD affects primarily Asians, it should be considered in the differential diagnosis of patients of any descent present with subcutaneous lesions accompanied by lymphadenopathy, peripheral eosinophilia and high serum IgE. The differential diagnosis, based on radiological findings, may include various neoplastic and inflammatory conditions such as soft tissue sarcoma, squamous cell carcinoma, lymphoma, cutaneous metastases, angioma, pyogenic granuloma, reactive lymphadenopathy, drug reactions, and Castleman’s disease.^1,3^ On the other hand, the list of histopathological differential diagnosis may consist of angiolymphoid hyperplasia with eosinophilia (ALHE), epithelioid hemangioendothelioma, angiosarcoma, and a reaction to arthropod bites.^1,3,12^ The entity commonly confused with KD is ALHE, which is a rare, vascular, inflammatory disease seen mainly in females during their third and fifth decade.^5^ ALHE is similar to KD but is generally regarded as a distinct entity. ALHE presents as firm, painless or pruritic, single or multiple nodules or plaques of varying colour in the skin or subcutaneous tissue of the head and neck regions, specifically around the ear.^5^ Dermal lesions of ALHE easily bleed and are rarely accompanied by lymphadenopathy or peripheral eosinophilia.^5^ KD, in contrast, usually involves deeper tissues, such as lymph nodes, salivary glands, and the subcutaneous tissues, and is frequently accompanied by hypereosinophilia.^5^ On histological examination, ALHE is characterised by the proliferation of blood vessels with large and pleomorphic epithelioid endothelial cells associated with characteristic inflammatory infiltrate with few eosinophils. Fibrosis and eosinophilic infiltration are more common in KD than in ALHE.^6,12^ Furthermore, KD is a non-neoplastic chronic inflammatory disorder, whereas ALHE is considered a benign to low-grade neoplastic condition.^3,13^

Treatment modalities of KD are controversial and may include immunosuppressive therapy, radiotherapy, and surgical excision.^1^ Observation is acceptable as a treatment modality if the lesions are neither symptomatic nor disfiguring.^1^ Oral corticosteroids are commonly used in patients with asymptomatic lesions, as in our patient who improved on oral steroid; however, the disease frequently recurs after the cessation of therapy. Further, topical corticosteroid treatment may be effective for localised disease.^1,13^ The course of this disease is chronic, and lesions are frequently persisting or recurring despite treatment. Untreated lesions, particularly in the head and neck, may lead to disfigurement secondary to the overgrowth.^13^ Smoking and systemic diseases are associated with poor prognosis and recurrence after treatment is common.^14^

## Learning points

1. KD may affect patients of any ethnicity and develop at unusual sites with a clinical presentation mimics a neoplastic process.
2. The disease should be considered in the differential diagnosis of patients of any descent present with mass lesions, particularly in head and neck, lymphadenopathy, peripheral eosinophilia and high serum IgE.
3. Clinical presentation of KD in the breast could include long-standing axillary lymphadenopathy and palpable lumps or diffuse breast enlargement without palpable masses.
4. The definitive diagnosis of KD can be only made by histopathological assessment of surgically excised lesions. Multiple lymphadenopathies, peripheral hypereosinophilia and high IgE levels, however, are also crucial findings for the diagnosis.
5. Radiological findings of breast KD include solitary or multifocal mass lesions and axillary lymphadenopathy, however, dermal and trabecular thickening and increased breast density without masses may be the only observed manifestations.
6. Absence of breast lump is an intriguing presentation in non-Asian descent KD of the breast.
7. Because of rarity of this condition in the breast, efforts to exclude malignancy are extremely important in patients with KD presenting with breast abnormality.
